# Full-wave modeling of broadband near field scanning microwave microscopy

**DOI:** 10.1038/s41598-017-13937-5

**Published:** 2017-11-22

**Authors:** Bi-Yi Wu, Xin-Qing Sheng, Rene Fabregas, Yang Hao

**Affiliations:** 1 School of electronic engineering and computer science, Queen Mary University of London, London, E14NS UK; 2 School of Information and Electronics, Beijing Institute of Technology, Beijing, 100081 China; 3 Institut de Bioenginyeria de Catalunya (IBEC), c/Baldiri i Reixac 11-15, 08028, Barcelona, Spain; 4 Departament d’Enginyeries, Electrónica, Universitat de Barcelona, C/Martí i Franqués 1, 08028 Barcelona, Spain

## Abstract

A three-dimensional finite element numerical modeling for the scanning microwave microscopy (SMM) setup is applied to study the full-wave quantification of the local material properties of samples. The modeling takes into account the radiation and scattering losses of the nano-sized probe neglected in previous models based on low-frequency assumptions. The scanning techniques of approach curves and constant height are implemented. In addition, we conclude that the SMM has the potential for use as a broadband dielectric spectroscopy operating at higher frequencies up to THz. The results demonstrate the accuracy of previous models. We draw conclusions in light of the experimental results.

## Introduction

Scanning microwave microscopy (SMM) is a near field scanning probe microscopy (SPM) technique that measures the local transmission of microwaves from a sample using a sharp probe close to the surface of sample. The SMM is a potential alternative to access the electromagnetic properties of samples such as electrical impedance and the complex permittivity with high spatial resolution. The SMM applications include the super-resolution imaging and characterization of inorganic and organic samples^1–14^, as well as the development of functional materials and devices at nanoscale such as molecular electronics^15^. In addition, the SMM has been applied to biological imaging for single bacterial^16^, live cells *in situ* ^17^, muscle cells^18^. In contrast to the scanning optical microscopy (NSOM), microwaves have larger penetration depths. Thus, the SMM provides a high-resolution mapping of both surface and internal properties of material samples. The frequency range commonly used by SMM is about 1 GHz to 20 GHz. However, in the last years the work of Lucibello *et al*.^6^, Trasobares *et al*.^15^. and Imtiaz *et al*.^10^. point out the capability of SMM operating at higher frequency bands. For example, the skin cancer detection at THz frequency^19^, the calculation of complex permittivity of water at millimeter-wave frequency band in biological tissues.

The SMM setup consists of a nano-sized sharp probe connected to a microwave source through an impedance matching circuit. The SMM provides an excellent spatial resolution down to the molecular and atomic scales compared to other near field microwave microscopy techniques^20–26^. SMM usually measures the S-parameter^1,4,8,11,18,27^, shifted resonant frequency^12^ or directly the complex impedance^5,9,28^ of tip-sample interaction. Then the capacitance or conductance can be calculated from the tip-sample system which reflects the dielectric property of sample. However, it is not so obvious to map these quantities in samples with local material properties such as complex permittivity or permeability. These quantities represent complex convolutions between the probe geometry and electromagnetic response including material properties and surface topography^20,28^. Due to the small interactions of the tip-sample system the SMM setup requires a high-sensitive and stable signal detection system. Thus, a good estimation of the tip-sample impedance is of great significance in the design of the impedance matching circuit, especially for future implementation of broadband SMMs. In addition, the full-wave modeling allows the study of SMM integrated with metamaterial component, such as a flat lens of negative refraction index that amplifies evanescent waves. Such lenses have been fully-studied and demonstrated at microwave frequencies^29,30^, and its integration with SMM would further improve the spatial resolution and may enhance the ability to detect buried features.

Our goal is the modeling of the SMM setup and their electrodynamic interaction with material samples at low and high microwave frequencies. We used the finite element method (FEM) for the numerical simulations. Our model takes into account the key factor of the so-called *low-frequency breakdown problem*. We point out that the quasi-static model provides a good accuracy in terms of calculating the tip-sample capacitance, while the calculation of dissipation loss becomes less accurate as the frequency goes higher. Also, we demonstrate that the quasi-static model and PEC approximation are accurate methods for calculating capacitance of the nano-sized probe tip operating at K-band or lower. Our simulations show that the SMMs operating at higher frequency provide better sensitivity on dielectric loss measurement while both field radiation and skin effect need to be taken into consideration. The proposed full-wave modeling of SMM will provide some physical insights for the development of broadband near field microwave scanning spectroscopy with high imaging precision by taking both propagation and evanescent wave components into consideration. This work will present a complete modal picture of SMM in line with the collection mode used in NSOM, which determine local material properties by applying the evanescent-scattering field to propagating field conversion and it may open up new frontiers of SMM research at higher frequencies up to THz.

In the next section, we shall write down the full-wave model derived form the Maxwell’s equations. This section starts by describing of the so-called *low-frequency breakdown problem*. Then is presented the equation for calculating the complex impedance of the tip-sample interaction derived from the wave equation for electric field. In addition, the finite element model for the governing wave equation is written down. We present a framework of high frequency applications linked to the SMM. We start the section of results by describing the geometrical parameters used in our simulations and comparing the quasi-static model with the full-wave model via an experimental approach curve. We discussed the model and the simulations in light of a number of experiments and applications. Finally, we draw conclusions on the proposed numerical modeling and the future directions for it.

### The model

The probe size of AFM is much smaller than the operating wavelength, and it barely radiates electromagnetic waves. In the local near field region, the electric field is nearly irrational and Maxwell’s equations are reduced to the equation of Poisson for electrostatic potential. The complex impedance of the tip-sample interaction can be calculated by solving the Poisson’s equation numerically. This approach has been widely used for modeling the SPM techniques at low frequencies (30–300 kHz) such as nanoscale capacitance microscopy^31^, electrostatic force microscopy (EFM)^32,33^ and SMM based applications^2,3,5,9,16,28^. In addition to the quasi-static models have been developed equivalent circuit for profiling doped semiconductors and measuring material conductivity^1,3,8^. The accuracy of these methods have been proved with analytical approximations for particularly shaped probes at low frequencies^31,32^. However, the scattering and magnetic field play no role in these previous models.

Evanescent fields are embedded in the solutions to the Maxwell ‘s equations, thus a full-wave approach is always preferred to model the near field microscopy, which accounts for all wave-numbers both real and imaginary. However, there exist significant challenges in the numerical modeling of nano-structures and nano-materials at microwave frequencies. Unlike near field imaging in the optical regime for NSOM applications where the tip-size is around $1/100\lambda$ at the infrared range^34,35^, or probes at millimeter scale for microwave applications^24–26^. The SMMs use an AFM probe with tip dimensions that can be less than $1/{10}^{6}\lambda$ at millimeter-wave frequency band^10^. This leads to the so-called *low-frequency breakdown problem* ^36,37^ for the full-wave numerical modeling of SMM. The key factor of this problem is the ill-conditioned system obtained for the finite element solution of Maxwell’s equations (see Supplementary Information S1).

The wave equation for electric field is derived from the Maxwell’s equations. Thus, in a domain ${\rm{\Omega }}$ the wave equation is given by

1 $$\nabla \times {({\mu }_{r})}^{-1}(\nabla \times {\bf{E}})-{\omega }^{2}{\varepsilon }_{0}{\mu }_{0}{\varepsilon }_{r}{\bf{E}}={\bf{J}},\quad {\bf{r}}\in {\rm{\Omega }}$$

where ${\varepsilon }_{r}={\varepsilon }_{r}^{^{\prime} }-j{\varepsilon }_{r}^{^{\prime\prime} }$ is the relative permittivity and ${\mu }_{r}={\mu }_{r}^{^{\prime} }-j{\mu }_{r}^{^{\prime\prime} }$ is the relative permeability. Given a current source **J** with intensity *I*_0_ the electric field **E** can be calculated by solving (1). Thus, the complex impedance $Z=R+jX$ for compute the tip-sample interaction can be calculated through the following equations

2 $$R=\frac{\omega }{|{I}_{0}{|}^{2}}{\iiint }_{V}{\varepsilon }_{0}{\varepsilon }_{r}^{^{\prime\prime} }{\bf{E}}{|}^{2}+{\mu }_{0}{\mu }_{r}^{^{\prime\prime} }{\bf{H}}{|}^{2}dV+\frac{1}{|{I}_{0}{|}^{2}}{\iint }_{S}Re[{\bf{E}}\times {{\bf{H}}}^{\ast }]\cdot d{\bf{s}}$$

3 $$X=\frac{\omega }{|{I}_{0}{|}^{2}}{\iiint }_{V}{\mu }_{0}{\mu }_{r}^{^{\prime} }|{\bf{H}}{|}^{2}-{\varepsilon }_{0}{\varepsilon }_{r}^{^{\prime} }|{\bf{E}}{|}^{2}dV$$

where $\omega$ is the angular frequency, *I*_0_ is the current intensity from tip apex to the substrate. The resistance impedance *R* corresponds to the electromagnetic energy loss including material dissipation (first term in (2)) and radiation or scattering (second term in (2)). The reactance corresponds to the electromagnetic energy stored in the near field region, $X > 0$ represents the dominated field is the magnetic field, and the reactance is inductive, while $X < 0$ represents the reactance is capacitive, and the electric field dominates.

To simplify the analysis and the simulation, we use a delta-gap source as the excitation port which is a common approach in antenna simulation using the Method of Moments (MOM)^38^. Namely, we set the current source **J** as an infinitely thin current line with constant intensity *I*_0_ connecting the probe cantilever and substrate which holds the sample under study. The numerical solution of (1) leads to the calculation of the electric and magnetic field. Therefore, the complex impedance can be computed by

4 $$Z=\frac{V}{{I}_{0}}$$

together with the equation for the voltage gap *V* between the probe surface and substrate

5 $$V={\int }_{L}{\bf{E}}\cdot d{\bf{l}}$$

where *L* is the path of the current source (see Supplementary Information S2). We use the finite element method to solve equation (1)^39^. Thus, we can excite the SMM probe electromagnetically without introducing the supporting circuits such as impedance transformer or coaxial line. Therefore, the multiscale problem in our numerical simulations is avoided. If we assume that the metallic tip-probe is a perfect conductor at microwave frequencies, we can use the perfect electric conductor (PEC) boundary condition on the probe surface ${{\rm{\Omega }}}_{probe}$ as follow

6 $$\hat{n}\times {\bf{E}}=\mathrm{0,}\quad {\bf{r}}\in {{\rm{\Omega }}}_{Probe}\mathrm{.}$$

In addition, we truncate the simulation domain ${\rm{\Omega }}$ using the following Sommerfeld boundary condition^40^

7 $$\hat{n}\times (\nabla \times {\bf{E}})-j{k}_{0}\hat{n}\times ({\bf{E}}\times \hat{n})=0\quad {\bf{r}}\in {{\rm{\Omega }}}_{S}$$

where ${{\rm{\Omega }}}_{S}$ is the truncation boundary. The Sommerfeld boundary condition involves the non-radiation of electromagnetic energy into the simulation domain as well as the electric or magnetic fields are not zero on the truncated boundary due to the existence of electromagnetic radiation.

We are now at a position to consider the numerical implementation of solving the governing wave equation (1) together with the boundary conditions (6) and (7) for an arbitrary geometry and materials. Following the standard finite element method procedure, the resultant matrix equation in the frequency domain is

8 $${\bf{A}}(\omega )x(\omega )=b(\omega \mathrm{).}$$

Here the matrix ${\bf{A}}(\omega )$ is calculated by

9 $${\bf{A}}(\omega )={\bf{S}}-{\omega }^{2}{\bf{T}}+j\omega {\bf{R}}$$

where **S** is the stiffness matrix, **T** is the mass matrix and **R** is the conductivity- and boundary condition- related matrix. Expanding the unknown electrical field **E** using a vector basis function **N**, the entities of these matrices are assembled from their elemental contributions. In the SMM simulation at low frequencies, entries of ${\omega }^{2}{\bf{T}}$ associated with the tip are assumed negligibles due to finite machine precision^37^, and this introduces singularities to ${\bf{A}}(\omega )$, therefore the failure of solving the matrix equation (8) is called low-frequency breakdown problem. To solve this problem, we first extract the ill-conditioned submatrix ${{\bf{A}}}_{ss}$ from the system matrix **A**, and then find the inverse of ${{\bf{A}}}_{ss}$ by using a generalized eigenvalue decomposition. Finally, the inverse of **A** can be computed via the method of Schur-Complement^37,41^ (see Supplementary Information S1).

## Results

**Figure 1:**
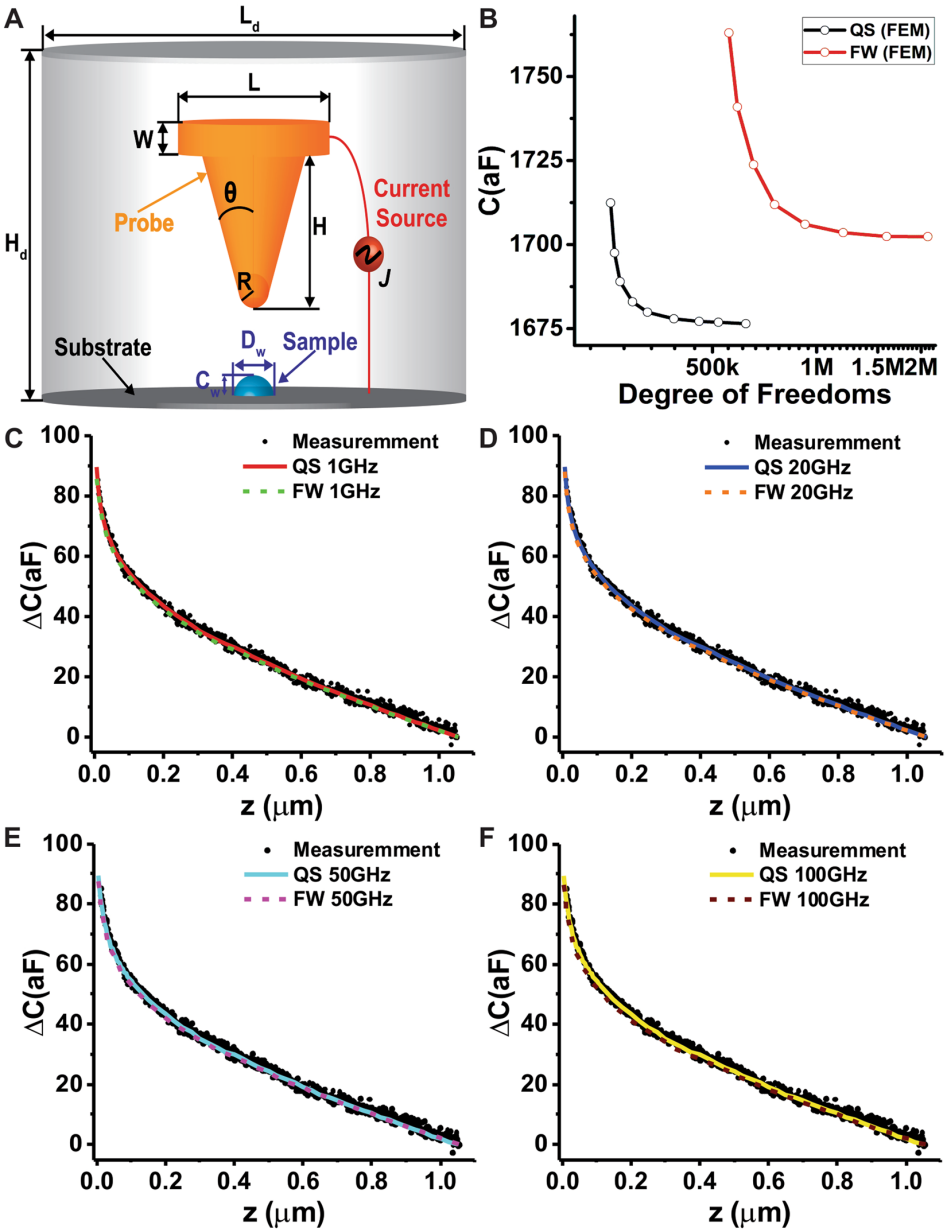
(**A**) Schematic representation of the model of SMM probe and the hemiellipsoid used in the numerical calculation (not to scale). The parameters used for the calculations: radius $R=217\,nm$, half cone angle $\theta ={10}^{\circ }$ and nominal values $H=40\,\mu m$, $W=6\,\mu m$ and $L=14.6\,\mu m$. (**B**) Mesh convergence test of the quasi-static (QS) and full-wave (FW) models as function of the degree of freedoms. (**C**–**F**) Theoretical capacitance gradient approach curves of the tip-substrate system of both models at different frequencies and the experimentally measured data nearly to 19 GHz.

The quantities used to determine the physical properties (relative permittivity) of a sample in the SMM measurement are the capacitance *C* and conductance *G* of tip-sample system from the admittance $Ys=1/Z=G+j\omega C$. Figure 1A shows the schematic three-dimensional metallic SMM probe with a sample placed on the conducting substrate. The tip is defined by a nose cone with a cylinder on top. The nose cone is a truncated cone of height *H* with half-angle $\theta$ and ended in a tangent spherical cap of radius *R*. *W* denotes the thickness of cylinder and *L* represents the diameter of cylinder.

### Example 1

The geometry dimensions of the SMM probe used in our calculations are: cone height $H=40\,\mu m$, $W=6\,\mu m$ and $L=14.6\,\mu m$. Here the tip radius $R=217\,nm$ and cone angle $\theta ={5}^{\circ }$ are calibrated parameters from the paper of Biagi, M.C. *et al*.^16^ (see Table. 2 in Supplementary Information). We show the FEM mesh convergence performance for the quasi-static and full-wave models in Fig. 1B. We solve the Poisson’s equation of the quasi-static model using a nodal basis function i.e. 10 degree of freedoms for a tetrahedral element. However, the vector basis functions i.e. 20 degree of freedoms for a tetrahedral element are required for solving the Maxwell ‘s equations in full-wave using FEM. Thus, the degree of freedoms for tetrahedral elements of full-wave FEM is generally larger than the required for the quasi-static FEM. The absolute capacitance is determined by the tip-sample interaction and the domain size, and it is also affected by the choice of truncation boundary condition. We eliminate the contribution of the domain size by considering the difference of capacitance ${\rm{\Delta }}C(x,y,z)=C(x,y,z)-C(x,y,{z}_{0})$ respect to a fixed point ${z}_{0}=1050\,nm$. Figure 1C–F depict the comparison of the theoretical approach curves calculated via the quasi-static model (using COMSOL AC/DC module) and the full-wave model, and the experimental measurement reported by M.C *et al*.^16^. The two theoretical models and the experimental data show a very good agreement which proofs the accuracy of the quasi-static approximation.

### Example 2

**Figure 2:**
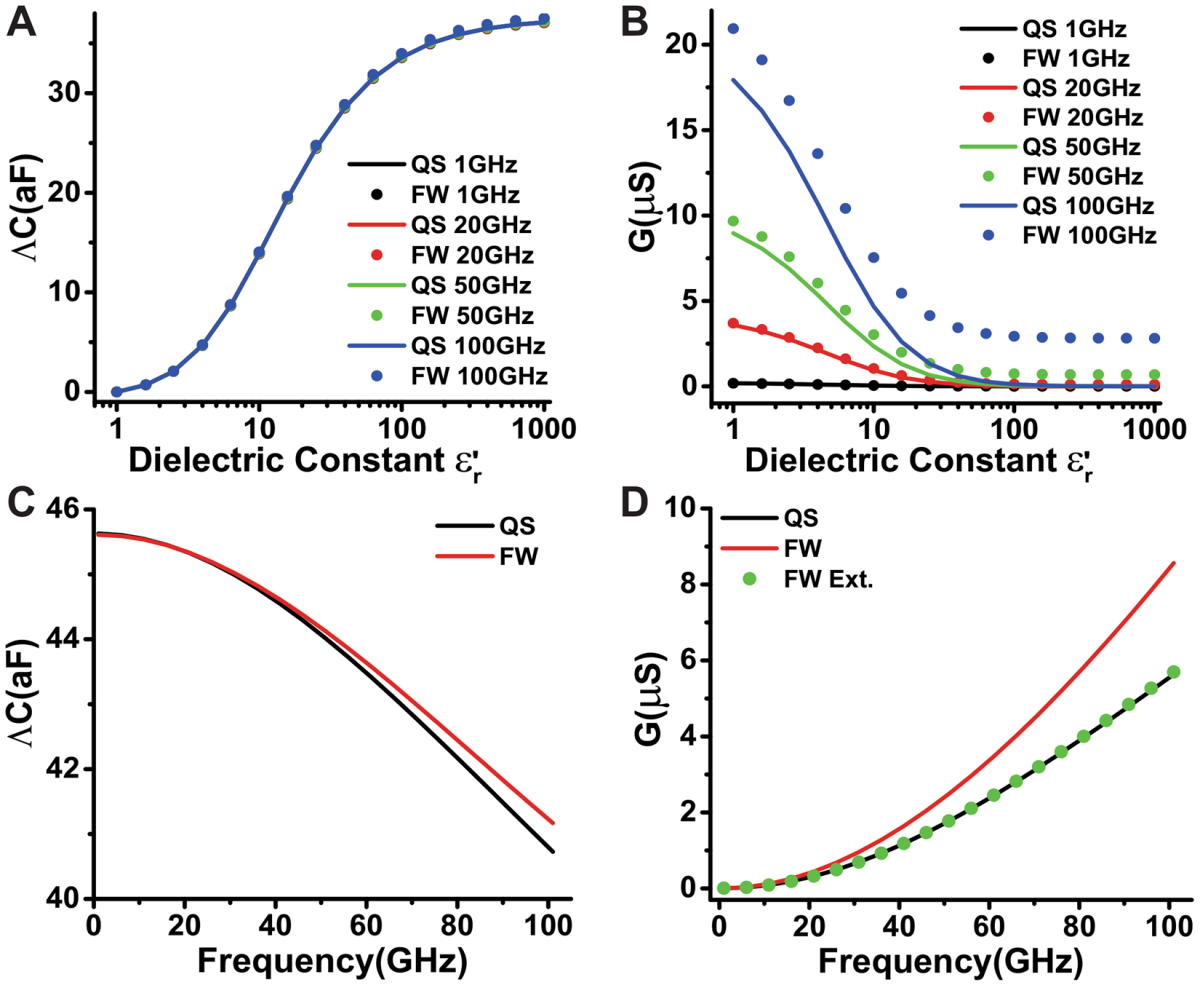
Contribution of a sample with hemiellipsoidal geometry that mimics a droplet of water by using the quasi-static model and full-wave method at different frequencies. Dimensions of the droplet of water: ${D}_{w}=1.0\,\mu m$ and ${C}_{w}=0.6\,\mu m$; relative permittivity of the sample is ${\varepsilon }_{{\rm{Sample}}}={\varepsilon }_{r}^{^{\prime} }-j{\varepsilon }_{r}^{^{\prime\prime} }$. (**A**,**B**) Intrinsic capacitance and total conductance as function of ${\varepsilon }_{r}^{^{\prime} }$ for fixed ${\varepsilon }_{r}^{^{\prime\prime} }=5.0$. (**C**,**D**) Intrinsic capacitance and conductance as a function of frequency for a dispersive sample.

The Fig. 2 compares the contribution of the quasi-static model and the full-wave model in capacitance and conductance for a sample with dimensions of ${D}_{w}=6\,\mu m$ and ${C}_{w}=2\,\mu m$. Here, ${D}_{w}$ and ${C}_{w}$ represent the axes of the hemiellipsoid depicted in Fig. 2A. This geometry mimics a droplet of pure water. The tip-sample distance for the calculations of fixed approach curve is 5*nm*. As in the previous example, the domain contribution is neglected by using the intrinsic capacitance ${\rm{\Lambda }}C(x,y,z)=C(x,y,z)-C({x}_{0},y,z)$ for ${x}_{0}\gg {D}_{w}$. Also, we assume that the sample is non-dispersive in the frequency band of interest to simplify the analysis. Later on we assume that the sample is dispersive and made of pure water whose dielectric constant is described by the modified Klein-Swift model with two Debye relaxations^42,43^.

Figure 2A,B shows the capacitance and conductance curves as function of the dielectric constant of the sample calculated by the two models. We point out that the intrinsic capacitance calculated via the two models almost match as shown in Fig. 2A. However, the computed conductance curves using the two methods are gradually separate from each other when the frequency increases as shown in Fig. 2B. This means that the radiation component dominates the total conductance when the frequency increases. The comparison of the intrinsic capacitance and total conductance for the two models are presented at Fig. 2C,D as function of the frequency. The intrinsic capacitance slowly decays due to the decrease of real part of the relative permittivity of pure water in the frequency range of 10 GHz to 100 GHz (see S3 in Supplementary information). We point out, that the difference between the results for the quasi-static model and the full-wave model becomes significant when the frequency increases. This implies that the frequency range for the accuracy of the quasi-static model is less than 30 GHz. At high frequencies, the local strength of magnetic field increases and the stored electric energy becomes smaller. Therefore, the intrinsic capacitance computed by the quasi-static model is smaller than full-wave model for the same ${\varepsilon ^{\prime} }_{r}$ (see Supplementary information S3 for absolute capacitances). In addition, the calculated conductance by the quasi-static model is smaller than the computed by the full-wave model. Furthermore, the radiation component in the total resistance impedance *R* increases when the frequency increases. The electromagnetic energy loss due to the material dissipation can be calculated via extracting the radiation contribution from the total conductance. The residual conductance is represented in Fig. 2D (curve of green dots). This curve matches very well with the result of quasi-static model.

The tip-sample system of SMM only stores electric energy because the size of tip is much smaller than the operating wavelength, and the capacitance is depend on the dielectric of the material. The quasi-static model is accurate for calculate the capacitance for sub-micron sized metallic tips operating below the K-band. However, for the characterization of dielectric loss, we have to use the full-wave model in order to take the radiation in consideration. The radiation is usually affected by the sample surface during the scanning procedure and it cannot be removed or calibrated. However, there might exist experimental techniques to overcome this problem, such as using a flat and ultra thin membrane to separate the sample and probe tip^17^.

### Example 3

**Figure 3:**
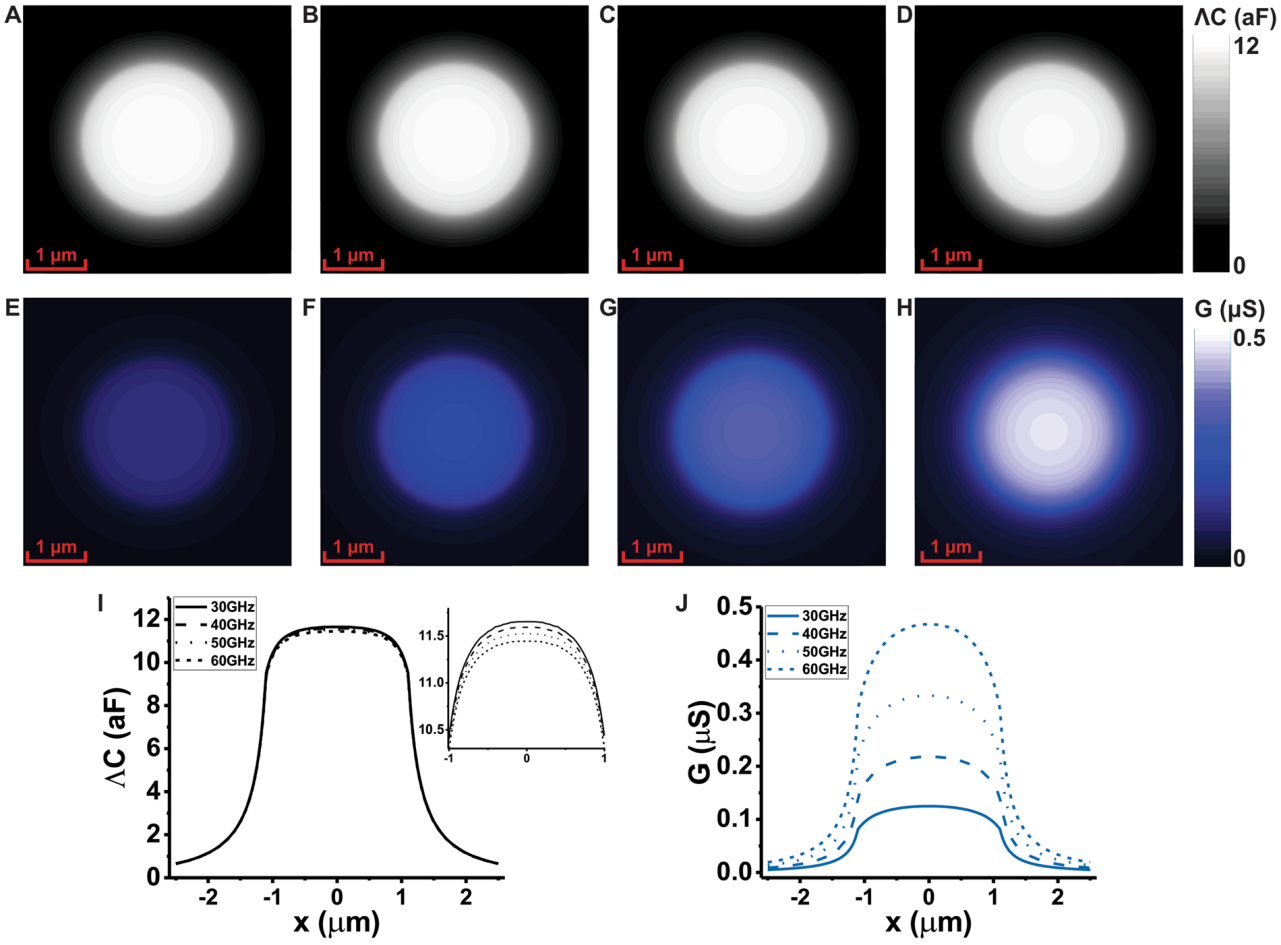
The SMM capacitance and conductance images of a water droplet at ${25}^{\circ }$. (**A**–**D**) intrinsic capacitance images at 30 GHz, 40 GHz, 50 GHz and 60 GHz. (**E**–**H**) Sample dissipation loss conductance images at 30 GHz, 40 GHz, 50 GHz and 60 GHz. (**I**–**J**) Numerically calculated cross-section intrinsic capacitance profiles and dissipation loss conductance profiles at 30 GHz, 40 GHz, 50 GHz and 60 GHz.

In this study, we apply the full-wave method to investigate a micron-size pure water droplet under SMM for the frequencies of 30 GHz, 40 GHz, 50 GHz and 60 GHz at constant height of 100 nm. We assume the same geometrical dimension as in the previous examples. The droplet of water is lying on a metallic substrate and is modeled as hemiellipsoid with semiaxes ${D}_{w}=1.0\,\mu m$ and ${C}_{w}=0.6\,\mu m$. Figure 3 shows the images and profiles in intrinsic capacitance and dissipation loss. Although ${\varepsilon }_{r}^{^{\prime} }$ of the sample decreases from about 27 at 30 GHz to 12 at 60 GHz (see S3 in Supplementary Information). The intrinsic capacitance image does not change significantly as shown in Fig. 3A–D and I. Figure 3E–H and J show the dissipation conductance images calculated from the radiation admittance of the total conductance. Here, the contrast increases due to the increase in frequency despite that ${\varepsilon }_{r}^{^{\prime\prime} }$ decreases from 33 to 21. This means that increasing the range of frequency of the SMM improve the image contrast related to the conductance/absorption of the sample.

The total conductance for the electric small probes is $G=Re\mathrm{(1}/Z)=R/({R}^{2}+{X}^{2})$. Here, the reactance is larger than the resistance impedance (.i.e $X\gg R$) and $|X| \sim 1/\omega C$. Thus, the total conductance of SMM small probe is about $G \sim R/{X}^{2} \sim RC{\omega }^{2}$. In this study, the capacitance is nearly constant over the frequency band of 30 GHz to 60 GHz and the decay of ${\varepsilon }_{r}^{^{\prime\prime} }$ is slower than $\mathrm{1/}{\omega }^{2}$. Therefore, the overall conductance increases as frequency increases. This shows that SMM operating at higher frequencies provide better contrasts in sub-wavelength imaging of lossy material samples. However, the accurate quantification of the dissipation requires the removal of the radiation loss contribution.

## Discussion

### Skin effect

The samples used in the SMM measurements usually are placed on a conducting surface (metals) and the tip-probe of AFMs is made of metals asuch as titanium, aluminum, tungsten^44,45^ or alloys^17^. At non-zero operating frequency, the electric current flowing in a conductor distributed towards the boundary due to the skin effect. The bulk skin depth of metals ranges from hundreds of nanometers to several micrometers at microwave frequency range which are comparable to the probe tip apex radius. For example, the bulk skin depth of titanium is 5.23 $\mu m$ at 5 GHz which is several times larger than the radius of the tip apex. Furthermore, the works of Vora *et al*.^46^ and G ü ney *et al*.^47^ have shown that the geometric skin depth of metallic nanostructures is much larger than bulk skin depth of the same conductor.

In previous studies of SMM and devices at low frequency such as EFM, the metallic components are assumed as perfect conductors with zero skin depth by applying a constant AC voltage to the boundaries. To validate the accuracy of this approximation, we simulated the metallic tip as a lossy medium in the full-wave model and compared the result with the perfect electric conductor (PEC) boundary model. Here, we assumed that the conducting substrate is an impedance boundary because its thickness is much larger than the skin depth, and the electromagnetic waves cannot penetrate the substrate^40^.

**Figure 4:**
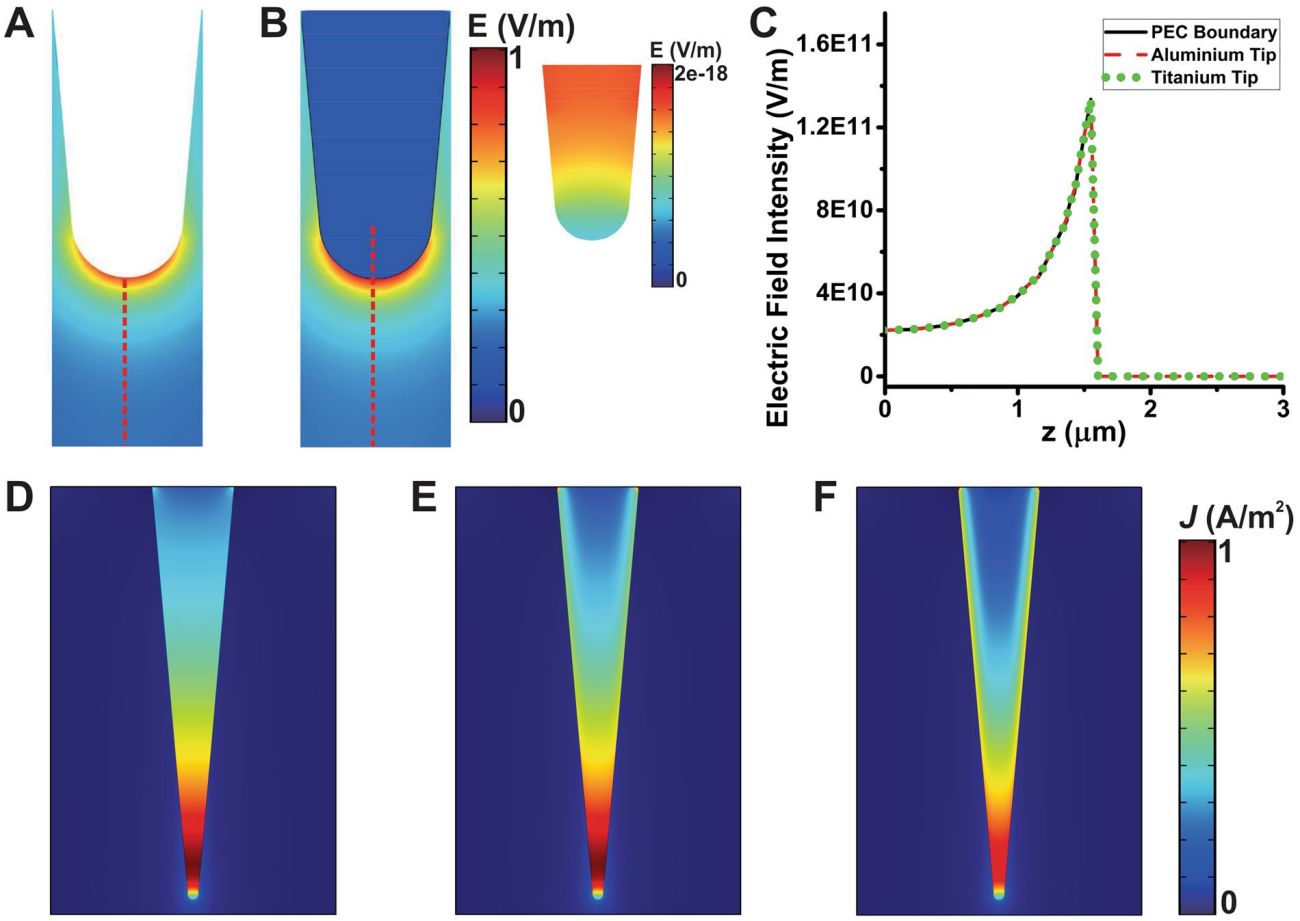
The electrical field of different types of probe tips at 5 GHz. (**A**) Normalized electrical field profile near the PEC boundary tip. (**B**) Normalized electrical field profile near the titanium tip. (**C**) Electric field intensity along the red dash lines in (**A**) and (**B**). (**D**–**F**) Normalized spatial current density in the cross-sections of different metal probe tip: (**D**) titanium tip; (**E**) tungsten tip; (**F**) aluminum tip.

The Fig. 4A,B presented the profile of normalized local electric field near a titanium tip modeled as the PEC and a lossy medium. We point out, that the profiles are quite similar, and the intensity of field inside the titanium tip modeled as a lossy medium is almost zero as shown the inset of Fig. 4B. In addition, Fig. 4C shows the electric field intensity along the red dash lines depicted in the Fig. 4A,B. Also, here is presented a very good agreement below the tip apex. The reflection coefficient from air to the probe surface is just less than unity due to the high metal conductivity. Thus, the incident energy is reflected by the metal tip such as the used probe PEC. Therefore, the field near the metallic probe is roughly identical to the field near the PEC probe. We also consider the case of metallic tips made of tungsten and aluminum, which have higher conductivity and smaller skin depth compared to the titanium. The normalized current density inside the titanium, tungsten and aluminum are shown in Fig. 4D,E. These non-magnetic metallic probe are simulated via the full-wave model as a lossy media with relative permittivity ${\varepsilon }_{r}={\varepsilon }_{r}^{^{\prime} }-j\sigma /\omega {\varepsilon }_{0}$. The conductivities of titanium, tungsten, and aluminum are 2.4e6S/m, 1.8e7S/m and 3.5e7S/m respectively. It seems that the geometric skin depth is larger for the metallic tip with a lower conductivity and the current penetrates into the tips of different materials.

**Figure 5:**
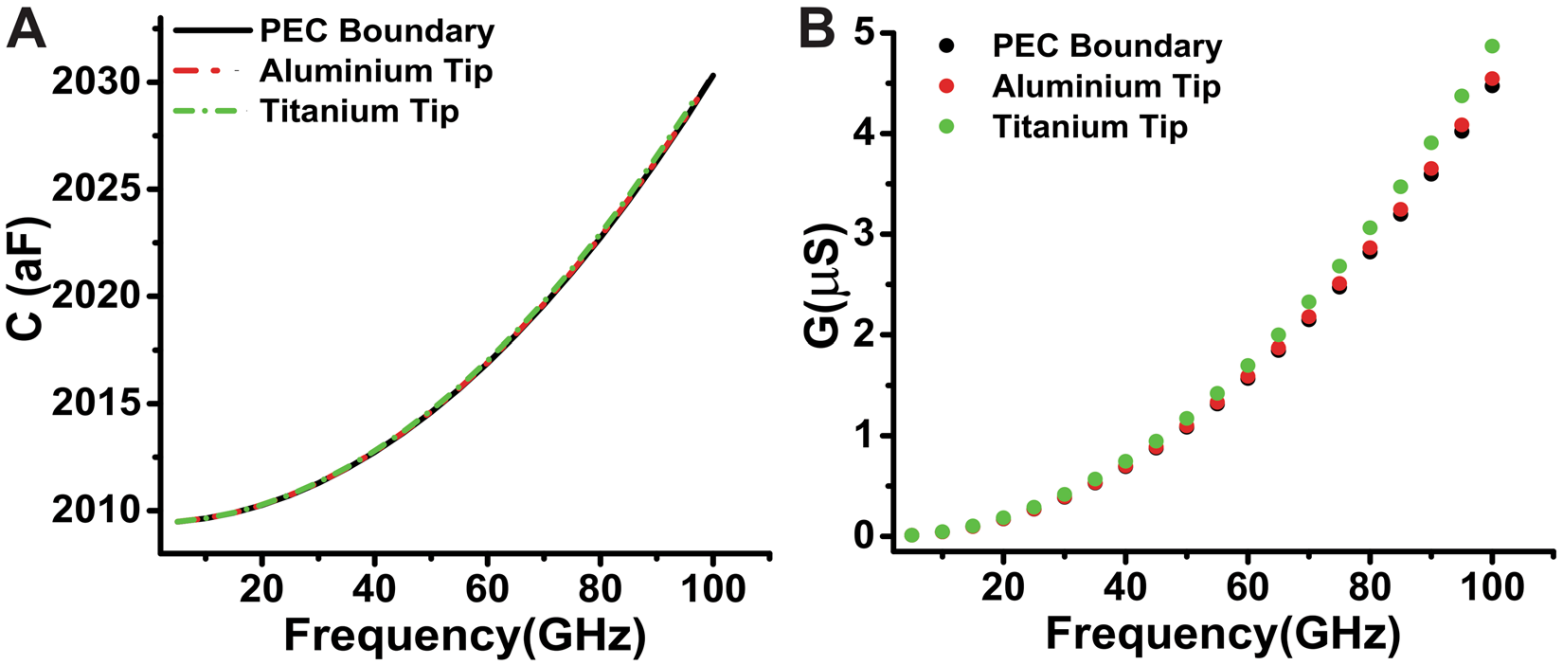
Numerical simulations of full-wave model for a sample with prism geometry that mimics a high-purity silicon sample of dimensions $8\,\mu m\times 8\,\mu m\times 1.6\,\mu m$ and different metallic probes. (**A**) Absolute capacitance and the total conductance as a function of the frequency.

Numerical results of metal tips placed on a micron-sized block of high purity silicon are shown in Fig. 5. In addition, the complex permittivity of high-purity silicon is reported in^48^ (see the S4 Supplementary Information). Figure 5A depicts the total capacitance of the tip-sample interaction calculated by the full-wave model using the PEC boundary condition and the lossy media with the conductivity of aluminum and titanium. There is no significant difference for the results of the three different probes. This implies, that the PEC boundary is a quite good approximation to the metallic tip. The skin effect has little effects on the capacitance values in SMM study. However, a notable differences between the PEC boundary and the lossy conductor simulations are presented in the total conductance results as shown in Fig. 5B. This means, that the electric currents penetrate the surface of the titanium and aluminum probes. Thus, the total conductance calculated for the titanium and aluminum probes is quite larger than the calculated for the PEC approximation. The radiation and sample dissipation loss in the total conductance under different probes are quite similar for the frequency range of 10 GHz to 100 GHz. However, in the same frequency band the dissipation losses are different (see S4 and Fig. 3 in Supplementary Information). This means, that the skin effect also a key factor to the calculation of total conductance at higher frequencies, in particular for probes made of metal with low conductivity.

### Metallic tip versus dielectric tip

**Figure 6:**
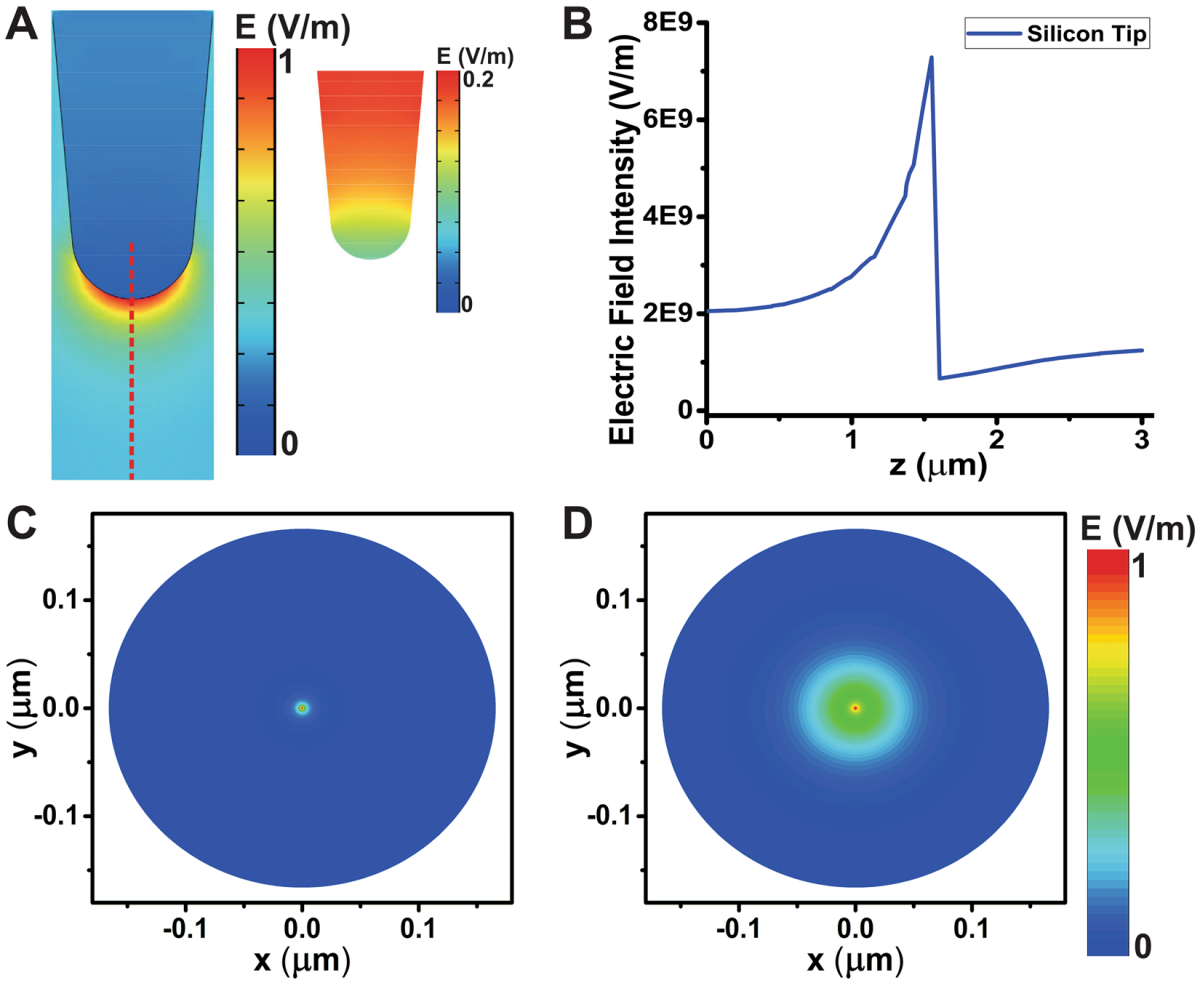
(**A**) Normalized electric field near and inside the high-purity silicon tip. (**B**) The electric field intensity on the red dash line. (**C**–**D)** Normalized electric field on a conducting substrate plane of radius 1.55 $\mu m$ below the SMM probe tip: (**C**) titanium tip, (**D**) silicon tip.

Some applications of the SMM use dielectric probes instead of metallic tips. For example, the silicon (Si) tip and silicon nitride (Si3N4) tip are commonly used in AFM^4^. The Fig. 6A shows the normalized electric field near and inside a silicon tip. The dielectric tip acting mimic a tapped dielectric wave-guide in NSOM. Figure 6B shows the intensity of electric field along the center line of tip to substrate (red line). The electric field near the silicon tip is less localized and the maximum intensity of the field is much lower compared to metallic probes. The electric field on a bare conducting substrate for a titanium tip and silicon tip with the same geometry is shown in Fig. 6C,D. The electric field under the metallic tip is more concentrated than under the silicon tip. This indicates, that the SMM with a metal tip has a better lateral resolution. The local electric field of the metallic tip might introduce a non-linear response of the samples. This means, that the dielectric probes could be a very good choice for certain applications.

## Conclusions

We presented a rigorous modeling of nanosized SMM probes and their electrodynamic interaction with material samples at microwave frequencies. We start by set out the full-wave model derived from the Maxwell’s equations for the SMM setup and taking into account the regularization of low-frequency breakdown problem in the numerical implementation. We demonstrated the accuracy of the models in a few examples by using the scanning techniques of approach curves and constant height. First, we demonstrated that the quasi-static model provides a quite good accuracy for the calculation of capacitance at low frequencies. While this model is less accurate for the dissipation loss for the higher frequencies. We also pointed out that the quasi-static model and the PEC boundary are good approximations for the theoretical calculation of capacitance. Here, our simulations show that the SMMs operating at higher frequency provide greater sensitivity on the evaluation of dielectric loss. Here, we shown that for accurate analysis the field radiation and skin effect need to be taken into consideration.

The numerical modeling presented here for the full wave model to mimics the SMM setup can be extended in a number of ways. First, the model can be used for the development of broadband near field microwave scanning spectroscopy with high imaging precision by taking into consideration the propagation and evanescent wave components. Another extension, is to use the model to determine local properties of materials by applying the evanescent-scattering field to propagating field conversion. This will enable to study new frontiers of SMM research at higher frequencies up to THz. A third extension is to embed the model in realistic physiological conditions (liquid media). This extension will enable the quantification of biological samples (bacteria, cells) in a realistic media.

## Electronic supplementary material

Supplementary information for full-wave modeling of broadband near field scanning microwave microscopy
