# Supplementary material for: Full-wave modeling of broadband near field scanning microwave microscopy
Source: Sci Rep. 2017 Nov 22;7:16064. doi: 10.1038/s41598-017-13937-5 (PMC5700110; doi:10.1038/s41598-017-13937-5)
Supplement: Supplementary file 1 — Supplementary information for full-wave modeling of broadband near field scanning microwave microscopy [file 41598_2017_13937_MOESM1_ESM.pdf]

Supplementary information for full-wave modeling of broadband near field scanning microwave microscopy

Biyi Wu, Xin-Qing Sheng, Rene Fabregas, and Yang Hao*

E-mail: y.hao@qmul.ac.uk

S1. Low-frequency breakdown problem

Using the full-wave model, the wave equation derived from Maxwell-equations is

$$\nabla \times \mu_r^{-1} (\nabla \times \mathbf{E}) - \omega^2 \epsilon_0 \mu_0 \epsilon_r \mathbf{E} = \mathbf{J} \tag{1}$$

where $\epsilon_r = \epsilon_r' - j\epsilon_r''$ and $\mu_r = \mu_r' - j\mu_r''$ are the relative permeability and permittivity respectively, and $\mathbf{J}$ is the source current. Following the standard FEM procedure, the discretized equation becomes to a matrix equation

$$\mathbf{A}(\omega)\{x(\omega)\} = \{b(\omega)\} \tag{2}$$

and the matrix $\mathbf{A}$ is the summation of

$$\mathbf{A}(\omega) = \mathbf{S} - \omega^2 \mathbf{T} + j\omega \mathbf{R} \tag{3}$$

where $\mathbf{S}$ is the stiffness matrix, and $\mathbf{T}$ is the mass matrices and $\mathbf{R}$ is the conductivity - and boundary condition- related matrix. They are assembled by the elemental contributions

$$\begin{aligned} \mathbf{S}_{ij}^e &= \int_{V^e} (\mu_r' - j\mu_r'')^{-1} (\nabla \times \mathbf{N}_i) \cdot (\nabla \times \mathbf{N}_j) dV \\ \mathbf{T}_{ij}^e &= \int_{V^e} \frac{\epsilon_r' - j\epsilon_r''}{c^2} \mathbf{N}_i \cdot \mathbf{N}_j dV \\ \mathbf{R}_{ij}^e &= \frac{1}{c} \int_{\Omega} (\hat{n} \times \mathbf{N}_i) \cdot (\hat{n} \times \mathbf{N}_j) dS \end{aligned} \tag{4}$$

and the right-hand side of (2) are assembled by

$$b_i^e = -j\omega\mu_0 \int_{V^e} \mathbf{N_i} \cdot \mathbf{J} dV \tag{5}$$

where $\mathbf{N}$ is the normalized vector basis function for electric field and V^e is the element volume in the simulation domain. Suppose the length of an element is l, the norm of $\nabla \times \mathbf{N}$ is proportional to $1/l$, and the norm of $\mathbf{S}_{ij}^e$ is the order of l and $\mathbf{T}_{ij}^e$ is the order of $10^{-17}l^3$. The ratio between $\mathbf{S}^e$ and $\omega^2\mathbf{T}^e$ is $10^{17}/\omega^2 l^2 \sim \lambda^2/36l^2$ and λ is the freespace wavelength. For a nanosized probe, to represent the mesh accurately, the minimum mesh size l can be as small as 1nm. Therefore, at microwave frequency band, $\omega^2\mathbf{T}^e$ is about 10^{15} times smaller than $\mathbf{S}^e$. The contribution of $\omega^2\mathbf{T}^e$ is treated as zero in $\mathbf{A}^e$ because of the round-off error in computing. As a result, the matrix $\mathbf{A}(\omega)$ becomes singular, and the full wave FEM solution breaks down. Generally, for the smaller tip, the low-frequency breakdown problem is more pronounced, because the minimum mesh size would be smaller.

To overcome this low-frequency breakdown problem due to the finite machine precision, we find the inverse of the nearly ill-conditioned component of matrix $\mathbf{A}$ by transforming it from a frequency dependent problem to a frequency independent generalized eigenvalue problem. To be more specific, we first divide the FEM matrix $\mathbf{A}$ into

$$\mathbf{A}(\omega) = \begin{bmatrix} \mathbf{A}_{ss}(\omega) & \mathbf{A}_{sr}(\omega) \\ \mathbf{A}_{rs}(\omega) & \mathbf{A}_{rr}(\omega) \end{bmatrix} \tag{6}$$

where $\mathbf{A}_{ss}$ represent the ill-conditioned submatrix, and it is associated with the region near the probe tip apex, $\mathbf{A}_{rr}$ is the regular component in $\mathbf{A}$. Correspondingly, the unknowns x in (2) are also divided into two categories: x_S and x_R. Here, $\mathbf{A}_{sr} = \mathbf{A}_{rs}^T$ represent the coupling between these two sets of unknowns.

According to the Schur-complement lemma, the inverse of $\mathbf{A}$ can be written as

$$\mathbf{A}(\omega)^{-1} = \begin{bmatrix} \mathbf{A}_{ss}^{-1} + \mathbf{A}_{ss}^{-1}\mathbf{A}_{sr}\mathbf{B}_{rr}^{-1}\mathbf{A}_{rs}\mathbf{A}_{ss}^{-1} & -\mathbf{A}_{ss}^{-1}\mathbf{A}_{sr}\mathbf{B}_{rr}^{-1} \\ -\mathbf{B}_{rr}^{-1}\mathbf{A}_{rs}\mathbf{A}_{ss}^{-1} & \mathbf{B}_{rr}^{-1} \end{bmatrix} \tag{7}$$

where

$$\mathbf{B}_{rr} = \mathbf{A}_{rr} - \mathbf{A}_{rs}\mathbf{A}_{ss}^{-1}\mathbf{A}_{sr} \tag{8}$$

here we omit the ω for simplicity. For SMMs operating in non-contact mode, the tip apex is surrounded by air which is lossless, the frequency dependency of $\mathbf{A}_{ss}$ can be written as

$$\mathbf{A}_{ss}(\omega) = \mathbf{S}_{ss} - \omega^2\mathbf{T}_{ss} \tag{9}$$

In FEM, matrices $\mathbf{S}_{ss}$ and $\mathbf{T}_{ss}$ are real and symmetric. Solving the following generalized eigenvalue problem

$$\mathbf{S}_{ss}\nu = \lambda\mathbf{T}_{ss}\nu \tag{10}$$

where λ is the eigenvalue and ν is the associated eigenvector, we have inverse of $\mathbf{A}_{ss}(\omega)$

$$\begin{aligned} \mathbf{A}_{ss}(\omega)^{-1} &= (V_0 V_h) \begin{bmatrix} -\omega^2\mathbf{I} & 0 \\ 0 & \Lambda_h - \omega^2\mathbf{I} \end{bmatrix}^{-1} (V_0 V_h)^T \\ &= -\frac{1}{\omega^2} V_0 V_0^T + V_h \left[\Lambda_h - \omega^2\mathbf{I}\right]^{-1} V_h^T \end{aligned} \tag{11}$$

In the right-hand side of (11), V_0 is the set of eigenvectors associated with zero eigenvalues, and represents the direct current (DC) modes near the tip apex area, and V_h is the set of eigenvectors associates with non-zero eigenvalues Λ_h. For SMM operating in contact mode

where tip touches the sample surface or surrounded by lossy media, the inverse of $\mathbf{A}_{ss}$ can be found in a similar way. Matrix $\mathbf{B}_{rr}$ usually is non-singular, and its inverse can be found normally or using eigenvalue decomposition again for a frequency dependent inversion . The electrical field in the simulation domain then can be calculated using the solved unknowns E and basis functions $\mathbf{N}$.

S2 Validation of the line port

To validate the correctness of the line port we used in this paper, we compare the complex impedance of the tip-sample interaction calculated by formula

$$Z = \frac{V}{I_0} \tag{12}$$

and their definitions (formula (1) and (2) in the paper). The value of dimensional parameters of the probe tip and simulation domain shown in Fig.1A of main text are given in Table.1 . The complex impedance of the probe tip 50nm above on bare substrate calculated by these two approaches agrees very well as shown in the Table.2. Here we use two kinds of basis functions in full-wave FEM: the linear basis functions and quadratic basis functions. The quadratic basis functions (with 20 DOFs for a tetrahedron) result in a much larger number of DOFs in the matrix equation, yet it has a higher accuracy comparing to the linear basis functions (with 6 DOFs for a tetrahedron). Thus we recommend using quadratic basis functions in the full wave FEM for SMM simulation.

Table 1: Geometric parameters of the SMM probe and the simulation domain

L_d	H_d	L	W	H	θ	R
322.53μm	200μm	18.52μm	6μm	40μm	10°	217nm

Table 2: Complex impedance calculated by full-wave FEM

Example	DOFs	$Z_{\text{port}}(\Omega)$	$R(\Omega)$	$X(\Omega)$
20GHz-Quadratic	760756	2.0148-j4510.4940	2.0129	-4510.4941
20GHz-Linear	116222	2.0830-j4347.3978	2.1490	-4347.3999

S3.The absolute capacitance of the dispersive sample

The dielectric constant of pure water at microwave frequencies calculated by modified Klein-Swift model from reference (38) is shown in Figure.1A . The absolute capacitances of the tip-sample interaction with and without the dispersive sample are shown in Figure.1B and Figure.1C, the intrinsic capacitance ΛC of these two figures are presented in Fig.2E of the main text. The absolute capacitance in Figure.1B is calculated by quasistatic model, while the counterparts in Figure.1C are calculated by full-wave model. The tip capacitance without sample calculated by quasistatic model is a constant value because the air in computation domain is non-dispersive. However, the capacitance for a tip on the bare substrate from 1GHz to 100GHz remain constant is unphysical. These two sets FEM calculations use the same sized simulation domain and the same mesh, while the absolute capacitances are different. This is because two different boundary conditions used in these two models. In the quasistatic model, the simulation domain is truncated by a surface on which the voltage is a constant value, thus both electric field and magnetic field are zero on this boundary. In our full-wave model, we use the Sommerfeld radiation boundary condition to truncate the simulation domain which enforces no energy radiated from infinity into the simulation domain. The electric and magnetic fields are non-zero on the boundary, and thus more electric charges are stored in the simulation domain comparing to those of quasistatic model, and the absolute capacitance of full-wave model is slightly larger.

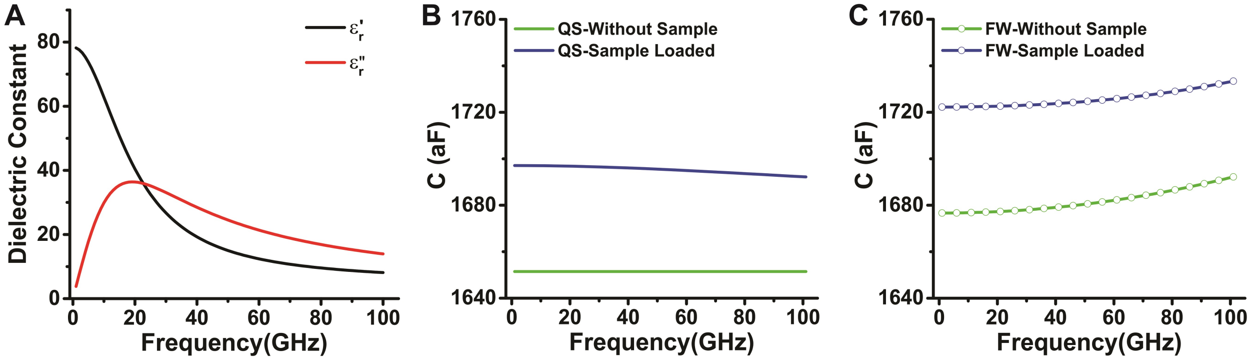

Figure 1: A.Dielectric constant of pure water at 25°C. B. Absolute capacitance of the tip with and without sample calculated by quasistatic model. C.Absolution capacitance of the tip with and without sample calculated by full-wave model.

S4 High-purity silicon sample under SMM and the skin effect

The dimension of the silicon sample is $8\mu m \times 8\mu m \times 1.6\mu m$, and this sample is placed 50nm under the probe tip apex as shown in Figure.2A. Other parameters including the geometry of probe tip and simulation domain size are given in Table.3. The real part of the relative permittivity of the high-purity silicon is almost a constant($\epsilon_r' = 11.6$) in the simulated frequency band, the conductivity of the sample is shown in Figure.2B.

Table 3: Geometry parameter of SMM probe tip and simulation domain

L_d	H_d	L	W	H	θ	R
332.03μm	200μm	28.03 μm	6μm	40μm	10°	517nm

The total conductance of the SMM tip-sample interaction in this study includes three parts: the radiation, the sample dissipation loss and the probe dissipation loss. The impedance

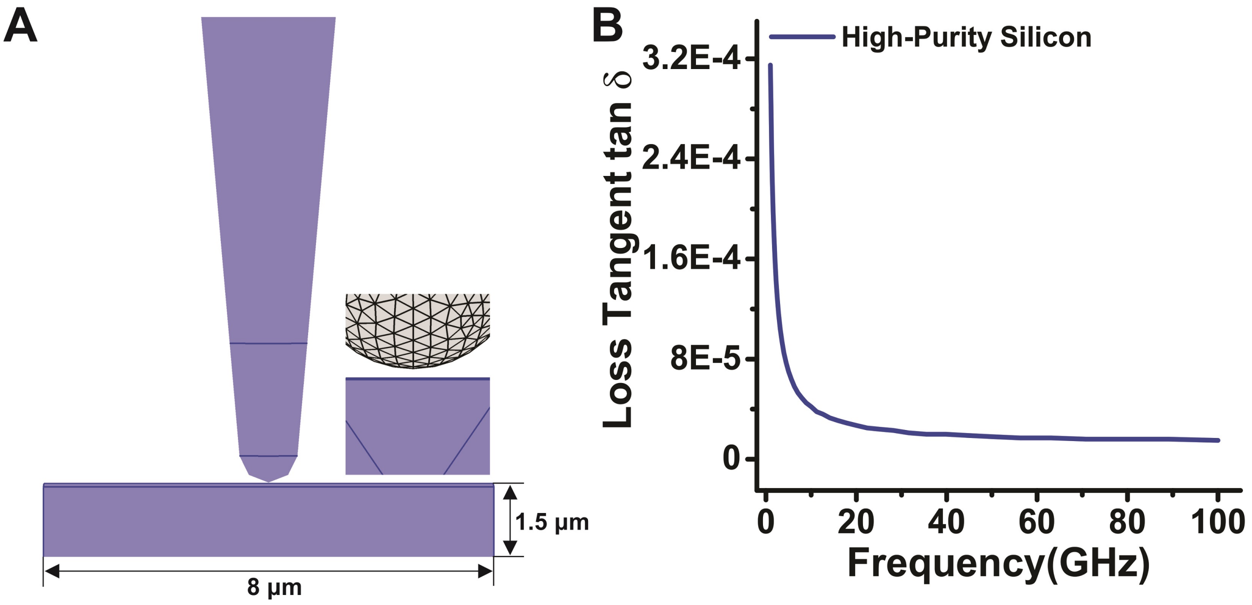

Figure 2: A highly pure silicon block under SMM probe. A.Geometry of the sample under the probe tip. B. The loss tangent of the silicon sample.

of these three parts are

$$\begin{aligned} R_{radi} &= \frac{1}{|I_0|^2} \iint_S Re\left[\mathbf{E} \times \mathbf{H}^*\right] \cdot d\mathbf{s} \\ R_{sample} &= \frac{\omega}{|I_0|^2} \iiint_{V_{sample}} \epsilon_0 \epsilon_r'' |\mathbf{E}|^2 dV \\ R_{probe} &= \frac{\omega}{|I_0|^2} \iiint_{V_{probe}} \epsilon_0 \epsilon_r'' |\mathbf{E}|^2 dV \end{aligned} \tag{13}$$

The radiation impedance, sample dissipation loss impedance and probe dissipation loss impedance from 1GHz to 100GHz are given Figure.3A-C. The inner field inside the PEC probe is zero, thus R_{probe} is zero for PEC boundary simulation. The radiation and sample dissipation loss are almost the same for different conductive probes, while the probe dissipation losses are different because of the skin effect. The metallic probe is highly conductive, and wave impedance of metal and of the surrounding air are so different that the reflection coefficient is just less than unity. Therefore, most of the incident energy is reflected by the metallic probe, only a small fraction of it is absorbed. The conductivity of PEC is infinitely large and all incident energy is reflected. At 100GHz, the absorbed energy of titanium probe

is only 0.8% (0.24/2.74) of the total energy loss, thus the field distribution near the metallic probe are almost identical to that near the PEC probe. In this study, the high-purity silicon is semi-insulator, the sample dissipation loss is minimum comparing to the radiation and probe dissipation loss as shown in this figure. This implies that it might be difficult to accurately characterize the local dissipation factor of high-purity silicon sample in SMM without considering the skin effects.

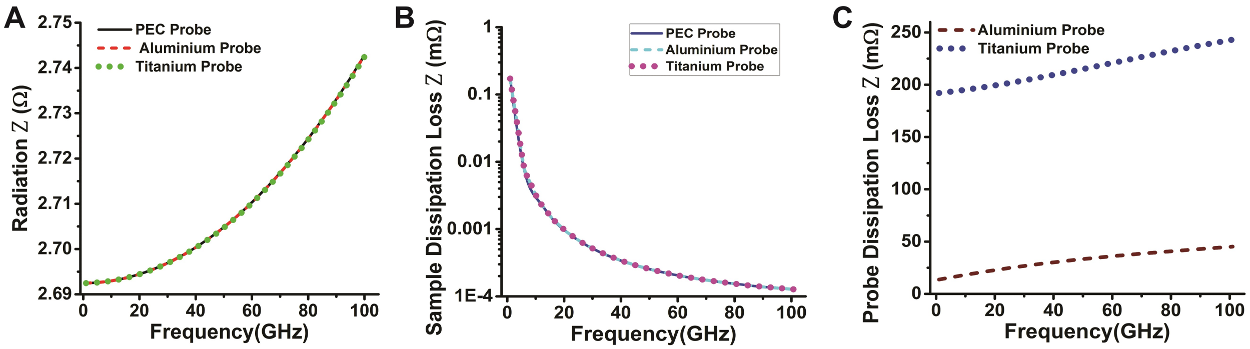

Figure 3: A.Radiation impedance of different kinds probes. B.Sample dissipation loss impedance of different kinds of probes. C.Probe dissipation loss impedance of different metallic probes.
